# Social Risks and Health Care Use in Medically Complex Patients

**DOI:** 10.1001/jamanetworkopen.2024.35199

**Published:** 2024-09-27

**Authors:** Emma L. Tucher, Allison L. Steele, Connie S. Uratsu, Jodi K. McCloskey, Richard W. Grant

**Affiliations:** 1 Division of Research, Kaiser Permanente Northern California, Oakland

## Abstract

**Importance:**

Integrated health care systems have initiated major investments to identify and address social risks, particularly for patients with multiple medical conditions.

**Objective:**

To evaluate the association of social risks with health care use among patients with complex multimorbidity.

**Design, Setting, and Participants:**

This longitudinal cohort study assessed Kaiser Permanente Northern California (KPNC) patients with (1) moderate medical complexity (defined by high comorbidity score, high risk of hospitalization, and/or prior emergency department [ED] admissions) and (2) high medical complexity (eg, meeting additional criteria, such as ≥7 medications and laboratory evidence of poor disease control).

**Exposure:**

Social risks (eg, requiring medical financial assistance and self-reported social barriers to care).

**Main Outcomes and Measures:**

Inpatient and outpatient health care use during 12 months of follow-up (January 15, 2023, to January 14, 2024).

**Results:**

The sample included 97 252 KPNC patients (mean [SD] age, 69.5 [16] years; 52.1% female; 10.6% Asian, 11.1% Black, 18.3% Hispanic, 54.6% White, and 5.5% other race or ethnicity [eg, American Indian or Alaskan Native, Native Hawaiian or Pacific Islander, multiracial, or unknown race]; and 8.8% insured by Medicaid), including 27 827 with moderate medical complexity (5074 [18.2%] with social risks) and 69 425 with high medical complexity (17 343 [25.0%] with social risks). In fully adjusted models, for moderate medical complexity, social risks were associated with higher odds of inpatient admissions (odds ratio [OR], 1.2; 95% CI, 1.1-1.4), ED visits (OR, 1.2; 95% CI, 1.1-1.3), and mental health visits (OR, 1.2; 95% CI, 1.1-1.3) vs individuals without social risks. Among individuals with high medical complexity, social risks were associated with higher odds of inpatient admissions (OR, 1.2; 95% CI, 1.1-1.2), ED visits (OR, 1.2; 95% CI, 1.1-1.2), and 30-day readmissions (OR, 1.2; 95% CI, 1.1-1.3) and higher odds of mental health visits (OR, 1.3; 95% CI, 1.2-1.3) vs individuals without social risks.

**Conclusions and relevance:**

In this cohort study of individuals with medical complexity, coexisting social risks were associated with substantial downstream health care use. Efforts to reduce use in individuals with complex medical comorbidity could include concurrent efforts to identify and reduce social risks.

## Key Points

**Question:**

How are social risks associated with 1-year inpatient and outpatient health care use outcomes in patients with complex medical needs?

**Findings:**

This cohort study of 97 252 patients with complex medical comorbidity found that social risks were associated with higher odds of inpatient admissions, emergency department visits, and mental health visits during a 1-year period. Individuals with added social risks were younger and more likely to be Medicaid eligible and Black or Hispanic.

**Meaning:**

Efforts to address health care use in patients with complex medical comorbidity may benefit from concurrent efforts to reduce social risks.

## Teaser

This cohort study of patients with complex medical comorbidity examines the association of social risks with inpatient and outpatient health care use outcomes.

## Introduction

A small subset of patients with multiple chronic conditions disproportionately drives US health care spending.^1,2^ This population with complex care needs is a major focus of health care systems, researchers, and policymakers seeking to improve health, reduce preventable health care use, and contain costs. An initial step toward addressing the high-need population is to identify key drivers of health care use.^3^

Researchers have segmented the high-need population based on medical characteristics: across studies, they tend to be older and manage disability, multiple chronic or major complex chronic conditions, have advancing illness, or experience frailty.^4,5,6,7,8,9^ Social risks are defined by factors such as financial strain, housing instability, and food insecurity, which can present barriers to effective health care.^10^ Recognizing the link between social risks and higher health care use and costs, worse disease outcomes, and shorter life expectancy, researchers have included social risks in high-need classifications.^11,12,13,14,15,16,17,18,19,20,21^ Studies^13,15,18,22,23,24^ quantifying the association of social risks with health care outcomes, use, and costs are often social risk or disease specific. Broader evidence on health care use is limited because many health care systems lack robust collection of patient social risks, leading to limited insights into their contribution to health care use.^3^

This study sought to examine the association of social risks with 1-year inpatient and outpatient health care use outcomes within a highly complex patient cohort. We used social risk characteristics, including neighborhood deprivation, visits with a social worker, and moving to a lower-resourced neighborhood, because they are readily captured in electronic health records (EHRs) and therefore broadly accessible to health systems to improve program targeting and design. We tested the hypothesis that patients with medical complexity and added social risks would have higher health care use than those without added social risks.

## Methods

### Setting and Participants

This cohort study was held within Kaiser Permanente Northern California (KPNC), an integrated health care delivery system with 4.3 million members with comprehensive capture of members’ EHRs, including inpatient and outpatient care within and outside the system. Kaiser Permanente Northern California provides health services to members insured through Medicaid, Medicare Advantage, the California health insurance exchange, and employer-based plans. Its members are representative of the Northern California population (ie, >30% of races other than White, 20% attained a high school degree or less, and 50% earn<$50 000 per year).^25^ This study was funded by the KPNC Delivery Science Fellowship Program. The Kaiser Permanente Institutional Review Board approved the study and granted permission for a waiver of consent for study participants as allowed under the Common Rule. This study followed the Strengthening and Reporting of Observational Studies in Epidemiology (STROBE) reporting guideline.

Beginning in 2017, KPNC piloted a care coordination program (Care Plus) for patients with complex medical and social risks at 6 medical facilities in Northern California. As part of the Care Plus program, a patient selection algorithm was developed and clinically validated to identify individuals with medical need and potentially addressable clinical and/or social factors. We applied this patient selection algorithm across KPNC to create an analytical cohort. We were interested in studying patients in the usual care setting and excluded participants in the Care Plus pilot program or with concurrent enrollment in a Medicaid care management program (n = 12 805). We excluded individuals without a mailing address in Northern California (n = 2168). Our analytic cohort included members with medical complexity from the 58 KPNC medical facilities not involved in the Care Plus pilot. The 12-month observation period spanned from January 15, 2023, to January 14, 2024.

### Classification of Medically Complex Population

We stratified the cohort into moderate medical complexity and high medical complexity based on the premise that social risks and strategies to address them would likely be different based on level of medical complexity. The moderate medical complexity cohort was defined as adult (aged ≥18 years) KPNC members with a high comorbidity burden (Comorbidity Point Score 2 [COPS-2] of 15-124),^26^ high risk for hospitalization (likelihood of hospitalization [LOH] in 6 months score >0.25),^26,27,28^ and/or 2 or more emergency department [ED] admissions in the prior year). The COPS-2, which ranges from 0 to 701, is a comorbidity score; higher scores indicate greater comorbidity. Our sample had scores between 15 and 124. With a logistic regression model applied to 12 months of historical clinical data, the LOH score estimates future admissions within 6 months. The LOH scores range from 0 to 1; higher scores indicate a higher likelihood of admission.

### Classification of Highly Medically Complex Population

The population with high medical complexity met criteria for moderate medical complexity and had 1 or more indicator of clinical complexity based on medication regimen (prescription of ≥7 concurrent medications, suboptimal adherence based on medication-dispensing records, prescription of high-risk medications defined by the Beers criteria, and/or chronic pain or dementia medications), chronic disease status (poor diabetes control with last hemoglobin A_1c_ level >10% [to convert to proportion of total hemoglobin, multiply by 0.01], kidney insufficiency with last glomerular filtration rate <30 mL/min, systolic heart failure with last ejection fraction ≤30%, and/or advanced lung disease with home oxygen), or geriatric risk (need for durable medical equipment for mobility and safety in the home, residential care facility admission in the prior year, albumin level <3.5 g/dL [to convert to grams per liter, multiply by 10], and/or diagnoses or self-report of weight loss, falls, fractures, or fall risk).

### Classification of Social Risks

Within both analytic cohorts, we identified individuals as having social risks if they met the following criteria. First, individuals were considered to have social risks if they had a high Neighborhood Deprivation Index^29^ or Social Vulnerability Index^30^ score (eg, they lived in a census tract within the top 2 quartiles of deprivation or vulnerability) plus at least 1 additional marker in their EHR associated with social risk. The social risk indicators were binary variables and included the following: seen by medical social worker in the prior year; received or applied for medical financial assistance coverage (as a proxy for financial strain); lived alone (as a proxy for social isolation); had 7 or more missed appointments in the past 6 months (as a proxy for potential barriers to health system engagement); moved from a home address in a higher-resourced community to a home address in a lower-resourced community as measured by the Neighborhood Deprivation Index and Social Vulnerability Index (indicating possible downward economic trajectory); or had 2 or more address changes in the prior year (as a proxy for housing instability and associated in KPNC with worse chronic disease control).^22^ Second, individuals were also considered to have social risks if they reported a social risk (eg, transportation barriers, housing instability, homelessness, food insecurity, social isolation, or financial insecurity) in response to the KPNC-administered Medi-Cal Integrated Outcomes Questionnaire or a Your Current Life Situation assessment^31^ (survey items available in eTable 1 in Supplement 1). More details on the patient selection criteria are available in eTable 2 in Supplement 1.

### Outcomes

We assessed health care use and mortality during the 12 months after cohort identification. We defined health care use as inpatient stays (including inpatient visits, observation visits, and 30-day readmissions), ED use (including total visits and visits that did not result in a hospitalization [ie, treat and release]), and outpatient visits (including primary care physician, specialty, mental health, and addiction medicine visits). We defined 30-day readmissions as all unplanned hospitalizations within a 30-day period after a preceding hospital discharge. We excluded same-day inpatient or observation discharges and those with a principal diagnosis of pregnancy or a condition originating in the perinatal period, involving patients who used a hospice benefit or who died or lost coverage during their stay. To account for the competing risk of insurance churn or death, we divided the total health care use counts by the months of follow-up available per patient. All health care use outcomes were count variables that summed each type of health care use across the number of patient-months.

### Additional Explanatory Variables

We included patient sex, age, self-reported race and ethnicity (Hispanic or Latino, non-Hispanic Asian, non-Hispanic Black, non-Hispanic White, or other [eg, American Indian or Alaska Native, Native Hawaiian or Pacific Islander, multiracial, or unknown race]), health insurance type (eg, Medicare Advantage or Medicaid), Medicare Advantage Special Needs Plan status, and Medicare and Medicaid dual eligibility status.^22^ Age, sex, and race and ethnicity are proxy variables for ageism, sexism, and structural racism, which impact health care use directly and indirectly through the social risks included in our patient selection algorithm (eg, food insecurity or housing instability).^32,33,34,35,36,37,38^ Similar to prior research, we included these variables as potential confounders in our analyses.^35^

### Statistical Analysis

We compared characteristics between individuals with and without social risks in both complex cohorts. We used the Pearson χ^2^ tests for binary and count variables. We fit separate multivariable logistic regression models with robust SEs to assess the association between added social risks and inpatient and outpatient health care use during the 12-month follow-up period. We then adjusted the multivariable logistic regression models for age, sex, race and ethnicity, health insurance type, dual eligibility, LOH score, ED count, and COPS-2 score. We included 2 sensitivity analyses. First, we used propensity score–matched inverse probability of treatment weights to adjust for baseline differences between individuals with and without social risks. An assessment of balance is available in eFigures 1 to 4 and eTable 3 in Supplement 1. Second, we analyzed the association of multiple exposures to social risk with health care use over time. All analyses were conducted using Stata, version 18 (StataCorp LLC). Two-sided *P* < .05 indicated statistical significance.

## Results

The sample included 97 252 KPNC patients (mean [SD] age, 69.5 [16] years; 52.1% female and 47.9% male; 10.6% Asian, 11.1% Black, 18.3% Hispanic, 54.6% White, and 5.5% other race or ethnicity [eg, American Indian or Alaskan Native, Native Hawaiian or Pacific Islander, multiracial, or unknown race]; and 8.8% insured by Medicaid): 27 827 with moderate medical complexity and 69 425 with high medical complexity. Of the 27 827 patients with moderate medical complexity (5074 [18.2%] with social risks), those with social risks were more likely to be female (56.1% with social risk vs 48.1% without social risk, *P* < .001), of ethnicity other than White (9.5% vs 10.9% Asian, 18.0% vs 10.8% Black or African American, 27.1% vs 19.6% Hispanic, and 5.6% vs 5.4% other races, *P* < .001 for all), significantly younger (mean [SD] age, 57.8 [19.0] vs 61.9 [18.0] years, *P* < .001), and Medicaid beneficiaries (15.3% vs 8.5%, *P* < .001) (Table 1). We identified 69 425 individuals with high medical complexity (17 343 [25.0%] with social risks); individuals with social risk were more likely to be women (57.9% with social risk vs 51.5% without social risk, *P* < .001), of ethnicity other than White (9.3% vs 11.0% Asian, 16.1% vs 8.8% Black or African American, and 22.9% vs 15.3% Hispanic, *P* < .001 for all), significantly younger (mean [SD] age, 71.1 [15.0] vs 73.4 [14.0] years, *P* < .001), dual eligible (6.2% vs 3.6%, *P* < .001), and Medicaid beneficiaries (13.3% vs 6.9%, *P* < .001) (Table 1).

**Table 1.** Sample Characteristics Across the Kaiser Permanent Northern California Medically Complex Cohorts, 2023^a^

Characteristic	Medical complexity, %					
	Moderate (n = 27 753)	Moderate with social risk (n = 5074)	*P* value	High (n = 52 082)	High with social risk (n = 17 343)	*P* value
Sex						
Female	48.1	56.1	<.001	51.5	57.9	<.001
Male	51.9	43.9		48.5	42.1	
Age, mean (SD), y	61.9 (18.4)	57.8 (19.4)	<.001	73.4 (13.6)	71.1 (14.9)	<.001
Age group, y						
18-29	6.7	9.9	<.001	1.0	1.6	<.001
30-49	17.9	23.7		4.9	7.3	
50-64	24.4	24.8		14.2	17.9	
65-74	22.3	19.4		27.4	27.4	
75-85	20.1	15.0		32.4	27.9	
≥85	8.5	7.3		20.1	18.0	
Race and ethnicity						
Asian, non-Hispanic	10.9	9.5	<.001	11.0	9.3	<.001
Black, non-Hispanic	10.8	18.0		8.8	16.1	
Hispanic	19.6	27.1		15.3	22.9	
White, non-Hispanic	53.3	39.7		59.5	45.9	
Other^b^	5.4	5.6		5.5	5.7	
Insurance type^c^						
Medicare	48.9	43.9	<.001	76.8	74.3	<.001
SNP	2.9	6.0	<.001	7.6	13.7	<.001
Medicaid	8.5	15.3	<.001	6.9	13.3	<.001
Dual eligibility	1.2	1.8	<.001	3.6	6.2	<.001
Clinical information						
COPS-2 score, mean (SD)	41.3 (23.8)	44.3 (25.6)	<.001	60.8 (28.4)	66.8 (29.2)	<.001
LOH score, mean (SD)	0.2 (0.2)	0.3 (0.2)	<.001	0.4 (0.2)	0.4 (0.2)	<.001
No. of ED admissions, mean (SD)	2.2 (1.8)	2.8 (3.0)	<.001	2.0 (1.8)	2.6 (2.7)	<.001

Abbreviations: COPS-2, Comorbidity Point Score; ED, emergency department; LOH, likelihood of hospitalization; SNP, Special Needs Plan.

^a^ Data are from Kaiser Permanente Northern California electronic health records, Your Current Life Situation and Medi-Cal Integrated Outcomes Questionnaire surveys, and Neighborhood Deprivation Index and Social Vulnerability Index.

^b^ Other race includes Native Hawaiian, Pacific Islander, American Indian, Alaska Native, multiracial, or unknown race.

^c^ Total does not sum to 100%; each insurance variable is a binary variable.

In the moderately medically complex cohort, individuals with social risks had higher baseline heath care use. At baseline, compared with those with no socials risks, they had higher inpatient admissions (mean [SD], 0.3 [0.9] vs 0.2 [0.7] visits per year; *P* < .001), ED visits (mean [SD], 2.0 [4.4] vs 1.3 [2.3] visits per year; *P* < .001), specialist visits (mean [SD], 4.4 [5.3] vs 3.8 [4.7] visits per year; *P* < .001), and mental health visits (mean [SD], 1.5 [5.9] vs 1.1 [5.1] visits per year; *P* < .001) (Table 2 and Figure). Similarly, individuals in the highly medically complex cohort with social risks had higher baseline health care use. At baseline, compared with those with no social risks, they had more inpatient stays (mean [SD], 0.5 [1.1] vs 0.4 [0.9] visits per year; *P* < .001), ED visits (mean [SD], 2.1 [3.5] vs 1.5 [2.4] visits per year; *P* < .001), primary care physician visits (mean [SD], 4.0[3.8] vs 3.5 [3.3] visits per year; *P* < .001), specialist visits (mean [SD], 5.0 [5.7] vs 4.6 [5.2] visits per year; *P* < .001), and mental health visits (mean [SD], 1.1 [5.1] vs 0.8 [4.5] visits per year, *P* < .001) (Table 2 and Figure). Addiction medicine visits were not associated with social risk status in either cohort (Table 2). Mortality during the 12-month follow-up period was highest in the highly medically complex cohort; individuals with concurrent social risks were more likely to die in the subsequent 12 months (9.6% vs 8.2%, *P* < .001); risk of death was not associated with social risk in the moderately medically complex cohort (2.7% vs 2.6%, *P* = .30) (Table 2). Churn during the 12-month follow-up period was higher in the moderately medically complex cohort; individuals with concurrent social risks were more likely to drop KPNC insurance in the subsequent 12 months (12.0% vs 8.9%, *P* < .001); in the highly medically complex cohort, this association also held (8.1% vs 6.1%, *P* < .001) (Table 2).

**Table 2.** Twelve-Month Inpatient and Outpatient Health Care Use Across the Kaiser Permanente Northern California Medically Complex Cohorts, 2023^a^

Variable	Medical complexity					
	Moderate (n = 22 753)	Moderate and social risk (n = 5074)	*P* value	High (n = 52 082)	High and social risk (n = 17 343)	*P* value
Death, %	2.5	2.8	.29	8.2	9.6	<.001
Churn, %	8.9	12.0	<.001	6.1	8.1	<.001
Inpatient visits, mean (SD), No.						
Hospitalization	0.2 (0.7)	0.3 (0.9)	<.001	0.4 (0.9)	0.5 (1.1)	<.001
Observation	0.1 (0.3)	0.1 (0.5)	<.001	0.1 (0.4)	0.2 (0.5)	<.001
ED	1.3 (2.3)	2.0 (4.4)	<.001	1.5 (2.4)	2.1 (3.5)	<.001
Treat-and-release ED	1.0 (2.0)	1.6 (4.0)	<.001	1.0 (2.0)	1.4 (3.0)	<.001
30-d Rehospitalization	0.0 (0.3)	0.1 (0.5)	<.001	0.1 (0.4)	0.1 (0.5)	<.001
Outpatient visits, mean (SD), No.						
Primary care physician	2.9 (2.9)	3.2 (3.5)	<.001	3.5 (3.3)	4.0 (3.8)	<.001
Specialist	3.8 (4.7)	4.4 (5.3)	<.001	4.6 (5.2)	5.0 (5.7)	<.001
Mental health	1.1 (5.1)	1.5 (5.9)	<.001	0.8 (4.5)	1.1 (5.1)	<.001
Addiction medicine	1.0 (7.8)	1.2 (9.7)	.052	0.4 (5.4)	0.4 (5.1)	.94

Abbreviation: ED, emergency department.

^a^ Data are from Kaiser Permanente Northern California electronic health records, Your Current Life Situation and Medi-Cal Integrated Outcomes Questionnaire surveys, and Neighborhood Deprivation Index and Social Vulnerability Index.

**Figure.**
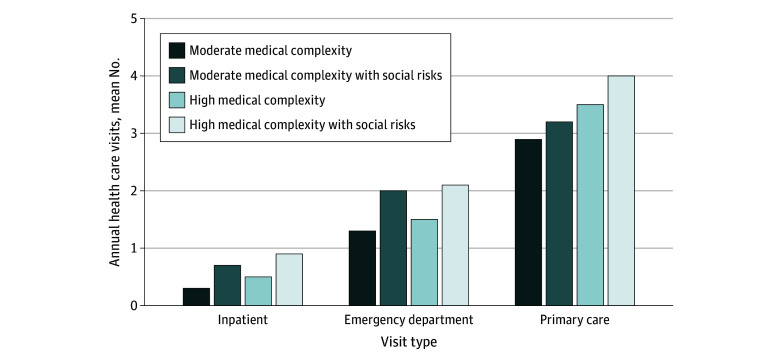
Annual Health Care Use Across 97 252 Individuals With Medical Complexity, 2023 Data are from Kaiser Permanente Northern California electronic health records, Your Current Life Situation and Medi-Cal Integrated Outcomes Questionnaire surveys, and Neighborhood Deprivation Index and Social Vulnerability Index.

In fully adjusted models, among individuals with moderate medical complexity, social risks were associated with higher odds of inpatient admissions (odds ratio [OR], 1.2; 95% CI, 1.1-1.4), ED visits (OR, 1.2; 95% CI, 1.1-1.3), treat-and-release ED visits (OR, 1.2; 95% CI, 1.1-1.3), and mental health visits (OR, 1.2; 95% CI, 1.1-1.3) during a 1-year period (Table 3). Among individuals with high medical complexity, social risks were associated with higher odds of inpatient admissions (OR, 1.2; 95% CI, 1.1-1.2), ED visits (OR, 1.2; 95% CI, 1.1-1.2), 30-day readmissions (OR, 1.2; 95% CI, 1.1-1.3), and mental health visits (OR, 1.3; 95% CI, 1.2-1.3) during a 1-year period (Table 3).

**Table 3.** Association Between Health Care Use and Social Risks, 2023^a^

Variable	Moderate medical complexity vs moderate medical complexity with social risks (n = 27 827)		High medical complexity vs high medical complexity with social risks (n = 69 425)	
	Unadjusted OR (95% CI)	Adjusted OR (95% CI)^b^	Unadjusted OR (95% CI)	Adjusted OR (95% CI)^b^
Inpatient visits				
Hospitalization	1.37 (1.27-1.48)^c^	1.24 (1.14-1.35)^c^	1.32 (1.27-1.37)^c^	1.17 (1.12-1.22)^c^
Observation	1.35 (1.21-1.50)^c^	1.23 (1.10-1.38)^c^	1.30 (1.24-1.37)^c^	1.16 (1.10-1.22)^c^
ED	1.42 (1.34-1.51)^c^	1.17 (1.09-1.25)^c^	1.40 (1.36-1.46)^c^	1.18 (1.13-1.22)^c^
Treat-and-release ED	1.44 (1.36-1.53)^c^	1.17 (1.10-1.25)^c^	1.35 (1.31-1.40)^c^	1.13 (1.09-1.18)^c^
30-d Readmissions	1.33 (1.10-1.62)^d^	1.07 (0.87-1.31)	1.50 (1.40-1.62)^c^	1.22 (1.13-1.32)^c^
Outpatient visits				
Primary care physician	0.93 (0.85-1.01)	0.97 (0.89-1.06)	1.01 (0.96-1.07)	0.97 (0.91-1.02)
Specialist	1.14 (1.05-1.22)^c^	1.09 (1.01-1.18)^e^	1.01 (0.97-1.06)	0.97 (0.93-1.01)
Mental health	1.45 (1.34-1.57)^c^	1.15 (1.06-1.26)^d^	1.43 (1.36-1.50)^c^	1.25 (1.18-1.32)^c^
Addiction medicine	1.05 (0.92-1.20)	0.82 (0.71-0.96)^e^	1.19 (1.07-1.34)^d^	0.97 (0.85-1.09)

Abbreviations: ED, emergency department; OR, odds ratio.

^a^ Data are from Kaiser Permanente Northern California electronic health records, Your Current Life Situation and Medi-Cal Integrated Outcomes Questionnaire surveys, and Neighborhood Deprivation Index and Social Vulnerability Index.

^b^ Adjusted analyses controlled for female sex, age, race and ethnicity, Medicare, Medicaid, Special Needs Program status, dual eligibility, Comorbidity Point Score 2 score, likelihood of hospitalization score, and number of ED visits.

^c^ *P* < .001.

^d^ *P* < .01.

^e^ *P* < .05.

In our sensitivity analysis using inverse probability of treatment weights to adjust for baseline differences between individuals with and without social risks, the results were similar in magnitude and direction as our main results. For the moderately medically complex cohort, social risks were associated with higher odds of inpatient admissions (OR, 1.2; 95% CI, 1.1-1.3), ED visits (OR, 1.2; 95% CI, 1.1-1.3), and mental health visits (OR, 1.2; 95% CI, 1.1-1.3) (eTable 4 in Supplement 1). For the highly medically complex cohort, social risks were associated with higher odds of inpatient admissions (OR, 1.2; 95% CI, 1.1-1.2), ED visits (OR, 1.2; 95% CI, 1.1-1.2), 30-day readmissions (OR, 1.2; 95% CI, 1.1-1.3) and mental health visits (OR, 1.3; 95% CI, 1.2-1.3) (eTable 4 in Supplement 1). In our sensitivity analysis in which we assessed social risk exposure as a count variable, we found that increased social risk exposure was associated with an increased magnitude of the incidence of inpatient and outpatient health care use across most types of health care use in both cohorts (eTable 5 in Supplement 1).

## Discussion

We evaluated the association between social risks and health care use among patients with medical complexity. Individuals with social risks were younger and more likely to be female, Medicaid eligible, and Black or Hispanic. In individuals with moderate and high medical complexity, having coexisting social risk was associated with significantly higher 1-year inpatient and outpatient health care use even after adjusting for demographic and clinical differences, including sex, age, race and ethnicity, health insurance type, and baseline health care use. Medical complexity is associated with patient burden, high financial burdens, and psychosocial challenges.^6,23,39,40,41,42,43^ We found that added social risks, which occurred in approximately 1 in 5 individuals in our sample, were associated with a substantially increased odds of hospitalization, ED use, and other health care use.

The higher odds of health care use observed among individuals with medical complexity and social risks may result from several factors. Social risks were more common in lower socioeconomic populations and associated with an increased risk of multimorbidity, frailty, and disability at any age.^37,44,45^ This finding aligns with prior research, which linked social factors (including structural racism and social risks) to a faster pace of biological aging, leading to the onset of medical complications at a younger chronological age.^37,44,45^ Our findings can be interpreted in the context of the broader research focused on health-related weathering and allostatic load, which posits the role of environmental factors in combination with clinical factors leading to downstream disease burden.^46,47,48^ We found an association between added social risks and higher mental health care use, which points to the potential importance of providing robust mental health support for these patients. This finding is salient because research shows improved emotional support underpins successful reductions in health care use linked to social risks.^49^

To address the increasing costs of high-need patients, health systems have invested in care management programs to coordinate care, connect individuals to resources, and provide health education and navigation with mixed results. Where effective, they are implemented in highly targeted, disease-specific, and regional contexts.^24,50,51,52,53,54,55,56,57,58^ Efforts to scale programs to larger populations have often failed to produce efficiencies or cost reductions.^59,60,61,62,63,64^ Most selection algorithms identify patients based on historical use, health diagnoses, or claims-based cost metrics; they often lack data on patients’ social risks.^50,54,56,57,63,65^ In this study, we found that social risks were associated with differences in the clinical and demographic picture of patients with medical complexity and therefore could likely be used to improve program selection and design. We also found that some characteristics that are readily captured in EHRs, including the Neighborhood Deprivation Index and Social Vulnerability Index, visits with a social worker, and moving to a lower-resourced neighborhood, can be used to identify social risks to improve program targeting and design. Health systems have an opportunity to leverage EHRs to document strategies, incorporate screening tools, and develop linkages to social services and community resources to better support medically and socially complex individuals.

### Limitations

This study has some limitations. Our results should be interpreted within the context of our study design. First, self-reported data on social risks, such as financial strain, food insecurity, and transportation barriers, were limited to individuals who completed the Medi-Cal Integrated Outcomes Questionnaire and Your Current Life Situation surveys. However, we identified additional social risks (eg, medical financial aid application and moving to lower income neighborhood) through access to KPNC’s comprehensive EHR and nationally available neighborhood characteristics databanks. Second, our data are based on KPNC’s EHRs, which limits generalizability to individuals with insurance and is confounded by variation in health-seeking behaviors across subpopulations. However, most adults older than 65 years in the US are insured (via Medicare), making our results largely generalizable. Our analysis is restricted to individuals insured in an integrated delivery system and residing within Northern California, which may limit generalizability. However, a recent study found that the demographics and measures of income, educational level, and social vulnerability of KP members are largely similar to those of nonmembers living in the same counties served by KP.^25^ Finally, we present data demonstrating an association between social risk and higher health care use but cannot determine causality based on our study design.

## Conclusions

In this cohort study, we identified a significant association of social risks with higher 1-year inpatient and outpatient health care use among patients with medical complexity within a single integrated health care system. These results can help inform policymakers and clinical leaders when planning social resource allocation and targeting, program expansions, and refining care management strategies. Our findings highlight that care management programs should consider social risks when designing programs. Ideal programs would address the care of older patients with the most medical complexity and multimorbidity and slow the advancement of morbidity in younger patients with less medical complexity. Future research should evaluate the role of social risks on accelerated biological aging and review the efficacy of potential interventions tailored to a socially complex, multiaged population.

## Notes

Supplement 1. **eTable 1.** Social Risks Survey Item Questions and Definitions **eTable 2.** Patient Selection Criteria **eFigure 1.** Propensity of Social Risk Exposure, Moderately Medically Complex, Balance Plot **eFigure 2.** Propensity of Social Risk Exposure, Highly Medically Complex, Balance Plot **eFigure 3.** Propensity of Social Risk Exposure, Standardized Differences, Raw and Weighted, Moderately Medically Complex **eFigure 4.** Propensity of Social Risk Exposure, Standardized Differences, Raw and Weighted, Highly Medically Complex **eTable 3.** Sample Characteristics Across the KPNC Medically Complex Cohorts With IPTW, 2023 **eTable 4.** Relationship Between Health Utilization and Social Risks With IPTW, 2023 **eTable 5.** Relationship Between Health Utilization and Social Risks by Number of Social Risk, 2023

Supplement 2.
Data Sharing Statement
