# Supplementary material for: Social Risks and Health Care Use in Medically Complex Patients
Source: JAMA Netw Open. 2024 Sep 27;7(9):e2435199. doi: 10.1001/jamanetworkopen.2024.35199 (PMC11437378; doi:10.1001/jamanetworkopen.2024.35199)
Supplement: Supplement 1. — eTable 1. Social Risks Survey Item Questions and Definitions eTable 2. Patient Selection Criteria eFigure 1. Propensity of Social Risk Exposure, Moderately Medically Complex, Balance Plot eFigure 2. Propensity of Social Risk Exposure, Highly Medically Complex, Balance Plot eFigure 3. Propensity of Social Risk Exposure, Standardized Differences, Raw and Weighted, Moderately Medically Complex eFigure 4. Propensity of Social Risk Exposure, Standardized Differences, Raw and Weighted, Highly Medically Complex eTable 3. Sample Characteristics Across the KPNC Medically Complex Cohorts With IPTW, 2023 eTable 4. Relationship Between Health Utilization and Social Risks With IPTW, 2023 eTable 5. Relationship Between Health Utilization and Social Risks by Number of Social Risk, 2023 [file jamanetwopen-e2435199-s001.pdf]

Supplementary Online Content

Tucher EL, Steele AL, Uratsu CS, McCloskey JK, Grant RW. Social risks and health care use in medically complex patients. *JAMA Netw Open*. 2024;7(9):e2435199. doi:10.1001/jamanetworkopen.2024.35199

eTable 1. Social Risks Survey Item Questions and Definitions

eTable 2. Patient Selection Criteria

eFigure 1. Propensity of Social Risk Exposure, Moderately Medically Complex, Balance Plot

eFigure 2. Propensity of Social Risk Exposure, Highly Medically Complex, Balance Plot

eFigure 3. Propensity of Social Risk Exposure, Standardized Differences, Raw and Weighted, Moderately Medically Complex

eFigure 4. Propensity of Social Risk Exposure, Standardized Differences, Raw and Weighted, Highly Medically Complex

eTable 3. Sample Characteristics Across the KPNC Medically Complex Cohorts With IPTW, 2023

eTable 4. Relationship Between Health Utilization and Social Risks With IPTW, 2023

eTable 5. Relationship Between Health Utilization and Social Risks by Number of Social Risk, 2023

This supplementary material has been provided by the authors to give readers additional information about their work.

© 2024 Tucher EL et al. *JAMA Network Open.*

eTable 1. Social Risks Survey Item Questions and Definitions

Social Risk	IOQ Question	YCLS Question	Positive Risk
Financial Strain	Please tell me whether in the past 3 months, you have had trouble paying for any of these things. (Select All that apply)<br><br>□ Healthy food (i.e., fruits and vegetables)<br>□ Housing<br>□ Heat and electricity<br>□ Medical needs<br>□ Transportation<br>□ Childcare<br>□ Debts<br>□ None of these<br>□ Other<br>□ Skip	In the past 3 months, did you have trouble paying for any of the following (Select ALL that apply)<br><br>□ Food<br>□ Housing<br>□ Heat and electricity<br>□ Medical needs<br>□ Transportation<br>□ Childcare<br>□ Debts<br>□ Other<br>□ None of these<br>□ Prefer not to answer	One or more checked (excluding Skip, None of these, and Prefer not to say)
Food Insecurity	In the past 3 months, how often have you worried that your food would run out before you had money to buy more?<br>□ Never<br>□ Sometimes<br>□ Often<br>□ Very often<br>□ Skip	In the past 3 months, how often have you worried that your food would run out before you had money to buy more?<br>□ Never<br>□ Sometimes<br>□ Often<br>□ Very often<br>□ Prefer not to answer	Sometimes, Often, or Very Often
	[Financial Strain question]		Healthy food or Food
Housing Barriers	Do you have any concerns about your current living situation, such as: housing conditions, ability to pay for housing or utilities, feeling safe, lack of more permanent housing, or something else?<br>□ Yes<br>□ No	Do you have any concerns about your current living situation, like housing conditions, safety, and costs?<br>□ Yes<br>□ No	Yes

© 2024 Tucher EL et al. *JAMA Network Open.*

	What is your current living situation? (Select ONE only)<br>□ Live alone in my own home (house, apartment, condo, trailer, etc.); may have a pet<br>□ Live in a household with other people<br>□ Live in a residential facility where meals and household help are routinely provided by paid staff (or could be if requested)<br>□ Live in a facility such as a nursing home which provides meals and 24-hour nursing care<br>□ Temporarily staying with a relative or friend<br>□ Temporarily staying in a shelter or homeless<br>□ Other - describe	Which of the following best describes your currently living situation?<br><br>□ Live alone in my own home (house, apartment, condo, trailer, etc.); may have a pet<br>□ Live in a household with other people<br>□ Live in a residential facility where meals and household help are routinely provided by paid staff (or could be if requested)<br>□ Live in a facility such as a nursing home which provides meals and 24-hour nursing care<br>□ Temporarily staying with a relative or friend<br>□ Temporarily staying in a shelter or homeless<br>□ Other<br>□Prefer not to answer	Temporarily staying with friend/relative OR Temporarily staying in shelter or homeless
	[Financial Strain question]		Housing
Lack of Transportation	Has lack of transportation kept you from getting to medical appts or medications?		Yes
	Has lack of transportation kept you from doing other things you need as part of daily living?		Yes
	[Financial Strain question]		Transportation

© 2024 Tucher EL et al. *JAMA Network Open.*

eTable 2. Patient Selection Criteria[a]

Medical Needs (N=97,252), mean (SD)		
Medical Complexity	COPS-2 score	56.5 (29.0)
	LOH	0.3 (0.2)
	ED visit count	2.2 (2.1)
**Clinical Needs (N=69,425), %**		
Medication Complexity	7+ medications	44.3
	Poor medication adherence	18.7
	High risk medications	5.4
	Dementia medications	4.4
	Chronic pain medications	0.6
Chronic Disease	DME order	36.6
	On home oxygen	9.6
	Last A1c>10%	2.7
	ETOH intoxication	2.6
	EF<=30%	1.3
	Last GFR >30	0.2
Geriatric Risk	Falls, fractures, fall risk	42.5
	Self-reported weight loss	17.4
	Albumin <3.5	13.0
	Frailty	8.8
	Residential care facility	1.6
**Social Risks (N=21,056), %**		
Social Risks[b]	NDI/SVI + visit with medical social worker	45.6
	NDI/SVI + MFA coverage or app	37.6
	NDI/SVI + 7+ FTKAs in prior 6 months	22.9
	Moved to less resourced community	14.7
	2+ address changes in prior year	2.4
	Social isolation	0.4
	Homelessness or housing instability	0.3

NOTE: NDI: Neighborhood Deprivation Index; SVI: Social Vulnerability Index; FTKA: failure to keep appointment; PCP: primary care provider; MFA: medical financial assistance; A1c: glycated hemoglobin; GFR: glomerular filtration rate; EF: ejection fraction; ETOH: ethyl alcohol; DME: durable medical equipment; neighborhood measurement was derived from the patient's address from the electronic medical record and analyzed at the census-tract level by quartile; transportation; [a]Totals do not sum to 100% as individuals can be part of multiple groups; [b]n<11 people have food insecurity, transportation barriers, or financial instability (along with another social risk criteria), redacted rows due to small samples

© 2024 Tucher EL et al. *JAMA Network Open.*

eFigure 1. Propensity of Social Risk Exposure, Moderately Medically Complex, Balance Plot

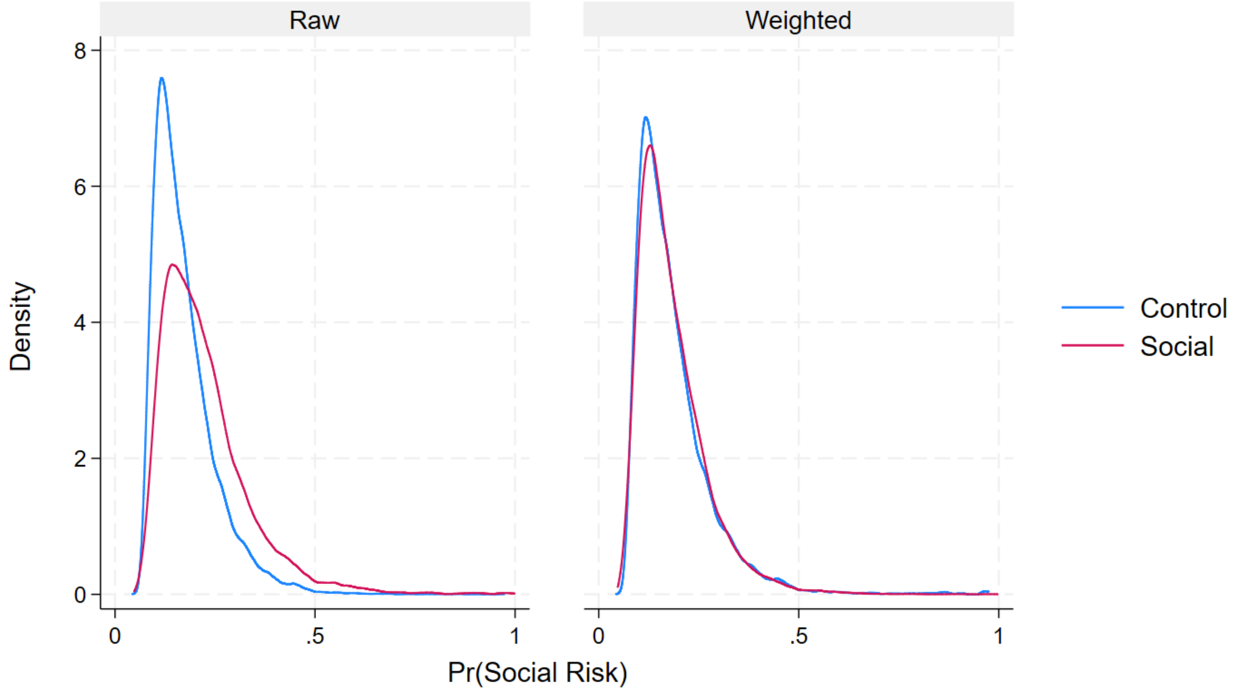

eFigure 2. Propensity of Social Risk Exposure, Highly Medically Complex, Balance Plot

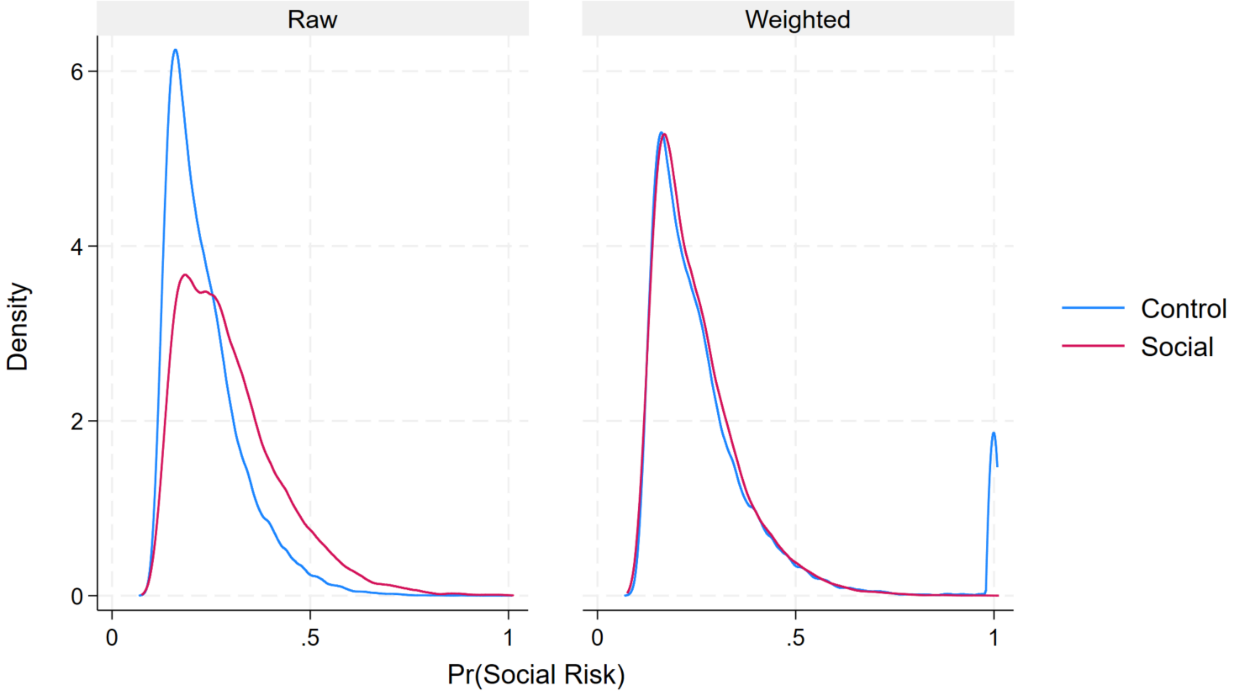

© 2024 Tucher EL et al. *JAMA Network Open.*

eFigure 3. Propensity of Social Risk Exposure, Standardized Differences, Raw and Weighted, Moderately Medically Complex

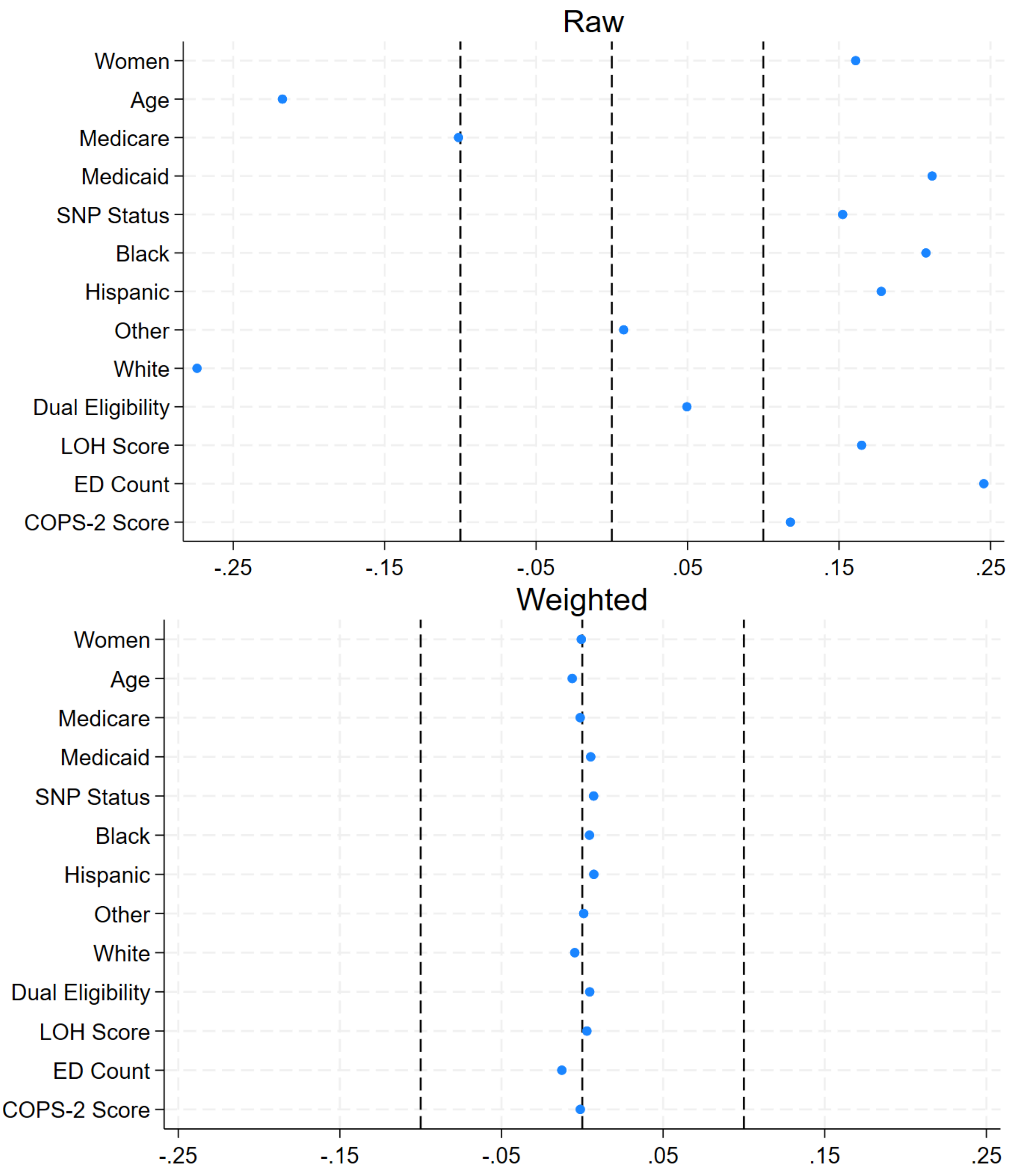

© 2024 Tucher EL et al. *JAMA Network Open.*

eFigure 4. Propensity of Social Risk Exposure, Standardized Differences, Raw and Weighted, Highly Medically Complex

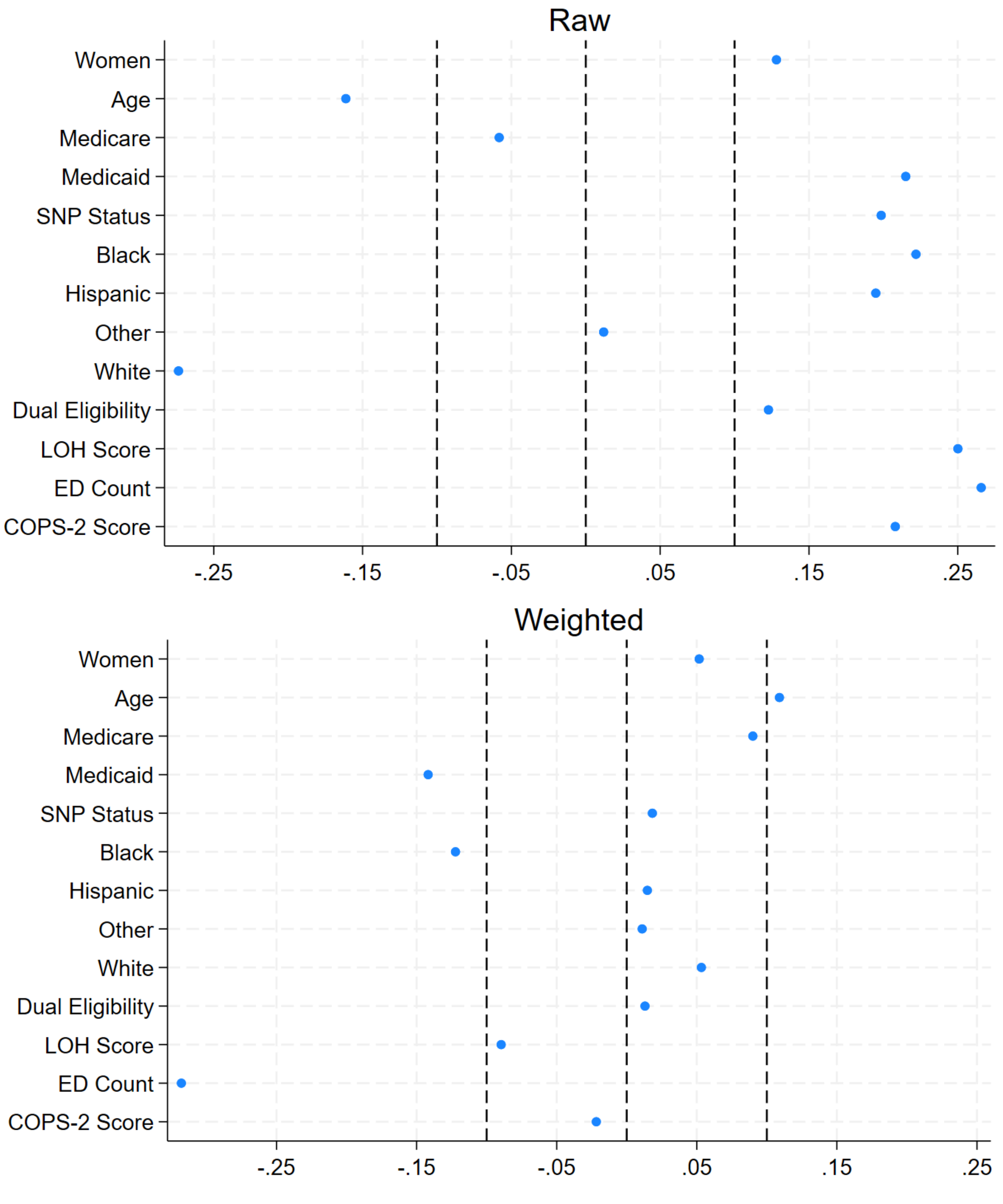

© 2024 Tucher EL et al. *JAMA Network Open.*

eTable 3. Sample Characteristics Across the KPNC Medically Complex Cohorts With IPTW, 2023

	Medical Complexity					
	Moderate	Moderate with Social	p-value	High	High with Social	p-value
Sample, N (%)	22,793 (81.9)	5,030 (18.1)	—	52,800 (76.1)	16,620 (23.9)	—
Women, %	49.5	49.6	0.97	50.9	53.5	0.26
Men, %	50.5	50.4		49.1	46.5	
Age, %						
Mean (SD)	61.1 (18.7)	61.0 (18.8)	0.71	71.3 (15.2)	72.9 (14.1)	0.16
18-29	7.5	7.0	—	1.2	1.1	—
30-49	18.8	20.3	0.02	10.2	5.6	0.24
50-64	24.3	25.3	0.06	14.1	16.0	<0.01
65-74	22.0	20.0	0.81	26.0	26.7	0.07
75-85	19.3	18.1	0.89	30.1	29.8	0.16
85+	8.1	9.4	<0.01	18.4	20.9	<0.01
Race/Ethnicity, %						
Asian, non-Hispanic	10.7	10.4	—	10.0	10.6	—
Black, non-Hispanic	12.1	12.3	0.62	14.7	10.6	0.17
Hispanic	21.0	21.3	0.57	16.8	17.3	0.54
Other[a]	5.5	5.5	0.77	5.3	5.5	0.80
White, non-Hispanic	50.8	50.5	0.77	53.2	55.9	0.76
Insurance Type, %[b]						
Medicare	47.9	47.8	0.94	72.4	76.3	0.19
SNP	3.5	3.7	0.62	8.7	9.3	0.27
Medicaid	9.8	9.9	0.72	13.1	8.7	0.13
Dual Eligibility	1.3	1.4	0.76	4.1	4.4	0.31
Clinical Information						
COPS-2, mean (SD)	41.9 (24.4)	41.9 (23.9)	0.94	63.2 (28.7)	62.6 (28.2)	0.53

© 2024 Tucher EL et al. *JAMA Network Open.*

LOH, mean (SD)	0.2 (0.2)	0.2 (0.2)	0.86	0.4 (0.2)	0.4 (0.2)	0.23
ED admissions, mean (SD)	2.4 (2.9)	2.3 (2.0)	0.59	6.3 (18.2)	2.1 (2.0)	<0.001

Source: KPNC Electronic Health Records, Your Current Life Situation and Medi-Cal Integrated Outcomes Questionnaire Survey data, Neighborhood Deprivation Index and Social Vulnerability Index data. NOTES: Sample N=84,249 participants in the study sample; adjusted analyses control for female, age, race/ethnicity, Medicare, Medicaid, SNP status, dual eligibility. * p<0.05, **p<0.01, ***p<0.001.

© 2024 Tucher EL et al. *JAMA Network Open.*

eTable 4. Relationship Between Health Utilization and Social Risks With IPTW, 2023

	Moderate Medical Complexity vs. Moderate Medical Complexity with Social Risks		High Medical Complexity vs. High Medical Complexity with Social Risks	
	Unadjusted	Adjusted	Unadjusted	Adjusted
Reference Group	Moderate Medical Complexity Only		High Medical Complexity Only	
Sample, N (%)	27,823 (28.6)		69,420 (71.4)	
Inpatient Visits, Odds Ratio (OR) (95% CI)				
Hospitalization	1.19 (1.10, 1.31)***	1.21 (1.11, 1.32)***	0.99 (0.73, 1.34)	1.18 (1.13, 1.23)***
Observation	1.17 (1.04, 1.32)**	1.19 (1.06, 1.34)**	0.83 (0.48, 1.45)	1.18 (1.11, 1.24)***
Emergency department (ED)	1.21 (1.13, 1.29)***	1.20 (1.12, 1.29)***	1.09 (0.94, 1.27)	1.19 (1.14, 1.24)***
Treat and release ED	1.21 (1.13, 1.29)***	1.20 (1.12, 1.29)***	1.03 (0.86, 1.23)	1.14 (1.10, 1.18)***
30-day Re-admissions	1.02 (0.82, 1.27)	1.05 (0.85, 1.31)	1.24 (1.09, 1.40)**	1.20 (1.11, 1.30)***
Outpatient Visits, OR (95% CI)				
Primary Care Provider	0.97 (0.89, 1.06)	0.98 (0.89, 1.07)	0.91 (0.81, 1.02)	0.96 (0.91, 1.02)
Specialist	1.08 (1.00, 1.17)*	1.09 (1.00, 1.18)*	0.90 (0.80, 1.01)	0.95 (0.91, 1.00)
Mental Health	1.18 (1.08, 1.28)***	1.19 (1.09, 1.31)***	0.87 (0.51, 1.49)	1.26 (1.20, 1.33)***
Addiction Medicine	0.86 74, 0.99)*	0.82 (0.70, 0.95)**	1.01 (0.87, 1.18)	0.98 (0.86, 1.11)

Source: KPNC Electronic Health Records, Your Current Life Situation and Medi-Cal Integrated Outcomes Questionnaire Survey data, Neighborhood Deprivation Index and Social Vulnerability Index data. NOTES: Sample N=84,249 participants in the study sample; adjusted analyses control for female, age, race/ethnicity, Medicare, Medicaid, SNP status, dual eligibility. Unadjusted results still reflect IPTWs. IPTWs account for female, age, race/ethnicity, Medicare, Medicaid, SNP status, dual eligibility, baseline LOH score, COPS-2 score, and ED count; * p<0.05, **p<0.01, ***p<0.001.

© 2024 Tucher EL et al. *JAMA Network Open.*

eTable 5. Relationship Between Health Utilization and Social Risks by Number of Social Risk, 2023

	Unadjusted	Adjusted
Reference Group	No social risks	
Sample, N (%)	84,249	
**Inpatient Visits, Incidence Rate Ratio (IRR) (95% CI)**		
Hospitalization		
1 social risk	1.30 (1.22, 1.39)***	1.14 (1.06, 1.22)***
2	1.82 (1.63, 2.02)***	1.30 (1.17, 1.45)***
3	2.09 (1.69, 2.60)***	1.26 (1.00, 1.58)
4+	0.58 (0.27, 1.23)	0.33 (0.15, 0.70)**
Observation		
1 social risk	1.27 (1.17, 1.37)***	1.13 (1.04, 1.23)**
2	1.70 (1.50, 1.93)***	1.25 (1.09, 1.42)**
3	2.31 (1.71, 3.12)***	1.35 (0.94, 1.95)
4+	0.88 (0.42, 1.84)	0.52 (0.25 ,1.08)
Emergency department (ED)		
1 social risk	1.37 (1.31, 1.43)***	1.16 (1.11, 1.22)***
2	1.75 (1.63, 1.89)***	1.18 (1.09, 1.27)***
3	2.28 (1.87, 2.77)***	1.15 (0.98, 1.35)
4+	4.02 (2.35, 6.88)***	1.94 (1.20, 3.14)***
Treat and release ED		
1 social risk	1.41 (1.34, 1.48)***	1.20 (1.14, 1.26)***
2	1.74 (1.61, 1.88)***	1.17 (1.06, 1.29)**
3	2.33 (1.81, 3.00)***	1.13 (0.91, 1.41)
4+	5.74 (3.25, 10.15)***	2.95 (1.83, 4.77)***
30-day Re-admissions		
1 social risk	1.70 (1.52, 1.92)***	1.10 (0.95, 1.27)
2	3.14 (2.73, 3.61)***	1.19 (0.95, 1.49)
3	3.30 (2.58, 4.21)***	1.15 (0.71, 1.87)
4+	0.72 (0.27, 1.94)	0.46 (0.13, 1.64)
**Outpatient Visits, IRR (95% CI)**		
Primary Care Provider		
1 social risk	1.16 (1.12, 1.19)***	1.08 (1.05, 1.12)***
2	1.42 (1.34, 1.51)***	1.24 (1.17, 1.32)***
3	1.54 (1.38, 1.72)***	1.25 (1.11, 1.41)***
4+	1.62 (1.18, 2.23)**	1.27 (0.93, 1.74)
Specialist		
1 social risk	1.08 (1.04, 1.12)***	1.04 (1.01, 1.08)*
2	1.39 (1.30, 1.47)***	1.24 (1.17, 1.32)***

© 2024 Tucher EL et al. *JAMA Network Open.*

3	1.68 (1.45, 1.94)***	1.41 (1.21, 1.32)***
4+	1.32 (0.97, 1.81)	1.08 (0.80, 1.47)
Mental Health		
1 social risk	1.24 (1.14, 1.36)***	1.13 (1.03, 1.23)*
2	1.67 (1.42, 1.96)***	1.43 (1.20, 1.71)***
3	2.44 (1.89, 3.14)***	1.99 (1.50, 2.62)***
4+	3.32 (1.57, 7.03)**	2.52 (1.12, 5.64)*
Addiction Medicine		
1 social risk	1.05 (0.78, 1.40)	0.89 (0.71, 1.12)
2	1.04 (0.70, 1.55)	0.93 (0.57, 1.50)
3	2.33 (1.25, 4.34)**	1.52 (0.65, 3.58)
4+	0.31 (0.05, 2.18)	0.41 (0.05, 3.72)

Source: KPNC Electronic Health Records, Your Current Life Situation and Medi-Cal Integrated Outcomes Questionnaire Survey data, Neighborhood Deprivation Index and Social Vulnerability Index data. NOTES: Sample N=84,249 participants in the study sample; adjusted analyses control for female, age, race/ethnicity, Medicare, Medicaid, SNP status, dual eligibility, high medical complexity, COPS-2 Score, ED count, and LOH score. * p<0.05, **p<0.01, ***p<0.001.

© 2024 Tucher EL et al. *JAMA Network Open.*
