# Supplementary material for: Social Risks and Health Care Use in Medically Complex Patients
Source: JAMA Netw Open. 2024 Sep 27;7(9):e2435199. doi: 10.1001/jamanetworkopen.2024.35199 (PMC11437378; doi:10.1001/jamanetworkopen.2024.35199)
Supplement: Supplement 2. — Data Sharing Statement [file jamanetwopen-e2435199-s002.pdf]

Data Sharing Statement

Tucher. Social Risks and Health Care Use in Medically Complex Patients. *JAMA Netw Open*. Published September 27, 2024. doi:10.1001/jamanetworkopen.2024.35199

Data
Data available: No

Additional Information
Explanation for why data not available: This data is only available to researchers from Kaiser Permanente Northern California and accessible after receiving KPNC's IRB approval.
